# Battling Recurrent Rhabdomyolysis in Carnitine Palmitoyltransferase II Deficiency

**DOI:** 10.7759/cureus.71524

**Published:** 2024-10-15

**Authors:** Muhammad Mohsin Isar, Hosna Ara Begum, Rafid Mustafa, Saifuddin Mohammad Kibria, Cornelius J Fernandez

**Affiliations:** 1 Acute Medicine, United Lincolnshire Hospitals NHS Trust, Boston, GBR; 2 Acute Medicine, United Lincolnshire Hospitals NHS trust, Boston, GBR; 3 Diabetes and Endocrinology, United Lincolnshire Hospitals NHS Trust, Boston, GBR

**Keywords:** acute kidney injury, carnitine palmitoyltransferase ii deficiency, creatinine kinase, long-chain fatty acid oxidation defect, rhabdomyolysis causing acute kidney injury

## Abstract

Carnitine palmitoyltransferase II (CPT II) deficiency is an inherited metabolic disorder that impairs the mitochondrial oxidation of long-chain fatty acids, leading to decreased utilization of these fatty acids. The adult form of this condition is characterized by recurrent rhabdomyolysis, often exacerbated by exercise, fasting, extremes of temperature, and infections. This case study details a severe episode of rhabdomyolysis triggered by a chest infection in a patient with CPT II deficiency. It discusses the management strategies for such acute episodes, emphasizing the importance of immediate intervention and avoiding potential triggers to prevent severe complications. The findings highlight the necessity of proactive management and preventive measures to mitigate risks in affected individuals.

## Introduction

Rhabdomyolysis is a serious condition resulting from the breakdown of muscle fibers, manifesting as muscle pain, weakness, and swelling. If not managed properly, it can lead to severe complications, including renal failure, liver dysfunction, cardiomyopathy, arrhythmias, and even death. One of the inherited causes of rhabdomyolysis is carnitine palmitoyltransferase II (CPT II) deficiency [1], which is an autosomal recessive disorder that impairs the ability of muscle cells to metabolize long-chain fatty acids for energy, particularly during prolonged exercise, resulting in energy depletion and muscle damage. Other inherited causes of rhabdomyolysis include various glycogen storage diseases, mitochondrial diseases, muscular dystrophies, and diseases involving abnormal intramuscular calcium handling [2].

CPT II deficiency can present in three forms: a lethal neonatal variant, a severe infantile variant, and a myopathic form that can occur at any time from infancy to adulthood. The myopathic form often requires additional external triggers, such as extended exercise, infections, fasting, or anesthesia, to precipitate an episode of rhabdomyolysis [3]. Effective management involves avoiding these triggers, early identification and treatment of symptoms, and addressing the underlying cause to prevent life-threatening complications. This paper presents the case of an adult male with recurrent severe rhabdomyolysis due to CPT II deficiency and discusses the complexities involved in managing his condition.

## Case presentation

A 65-year-old male with a history of CPT II deficiency, diagnosed since childhood, presented with a one-day history of productive cough, shortness of breath, and sore throat. He also reported feeling generally unwell and experiencing muscle pain in his arms and shoulders but denied any chest pain, orthopnoea, paroxysmal nocturnal dyspnea, or hemoptysis. His medical history is significant for type 2 diabetes mellitus (T2DM), chronic kidney disease (CKD), essential hypertension, and methemoglobinemia. His regular medications included amlodipine 5 mg twice daily (BD), Atorvastatin 10 mg nocte (ON), and ramipril 2.5 mg daily (OD), with diabetes managed through lifestyle changes.

Eight years earlier, he was admitted to our intensive therapy unit (ITU) for sepsis secondary to bilateral pneumonia. During that admission, he presented with flu-like symptoms, dry cough, and epigastric pain. His respiratory viral swabs came back positive for influenza A. His arterial blood gas (ABG) revealed type 2 respiratory failure (T2RF) with respiratory acidosis. Despite initial treatment with antibiotics and non-invasive ventilation (NIV), his condition deteriorated, necessitating intubation and invasive ventilation. Due to his CPT II deficiency, he developed rhabdomyolysis and acute kidney injury (AKI), requiring hemofiltration. During that episode, he also needed inotropic support and a prolonged ITU stay with a tracheostomy.

**Figure 1:**
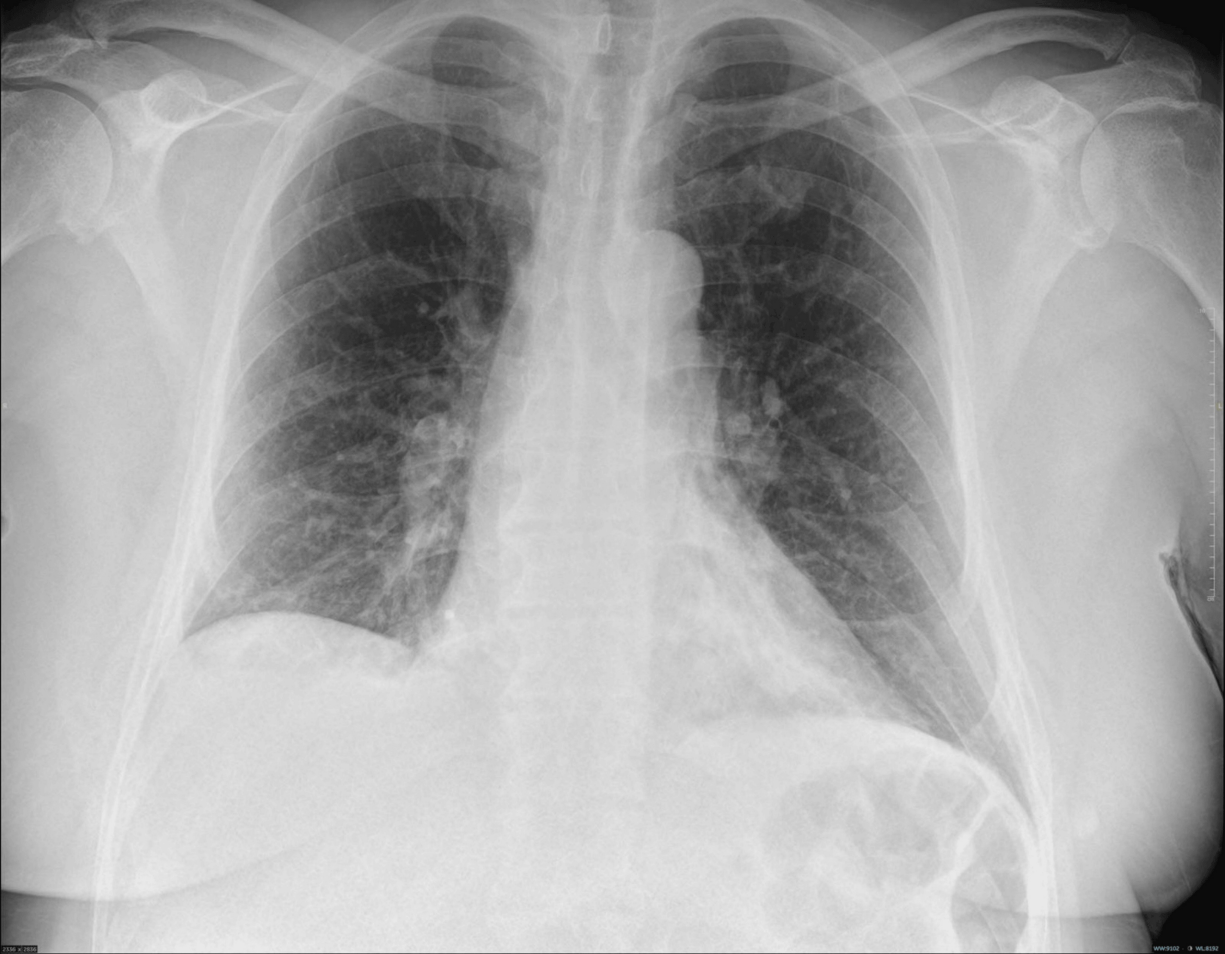
Chest X-ray during current hospital stay.

**Figure 2:**
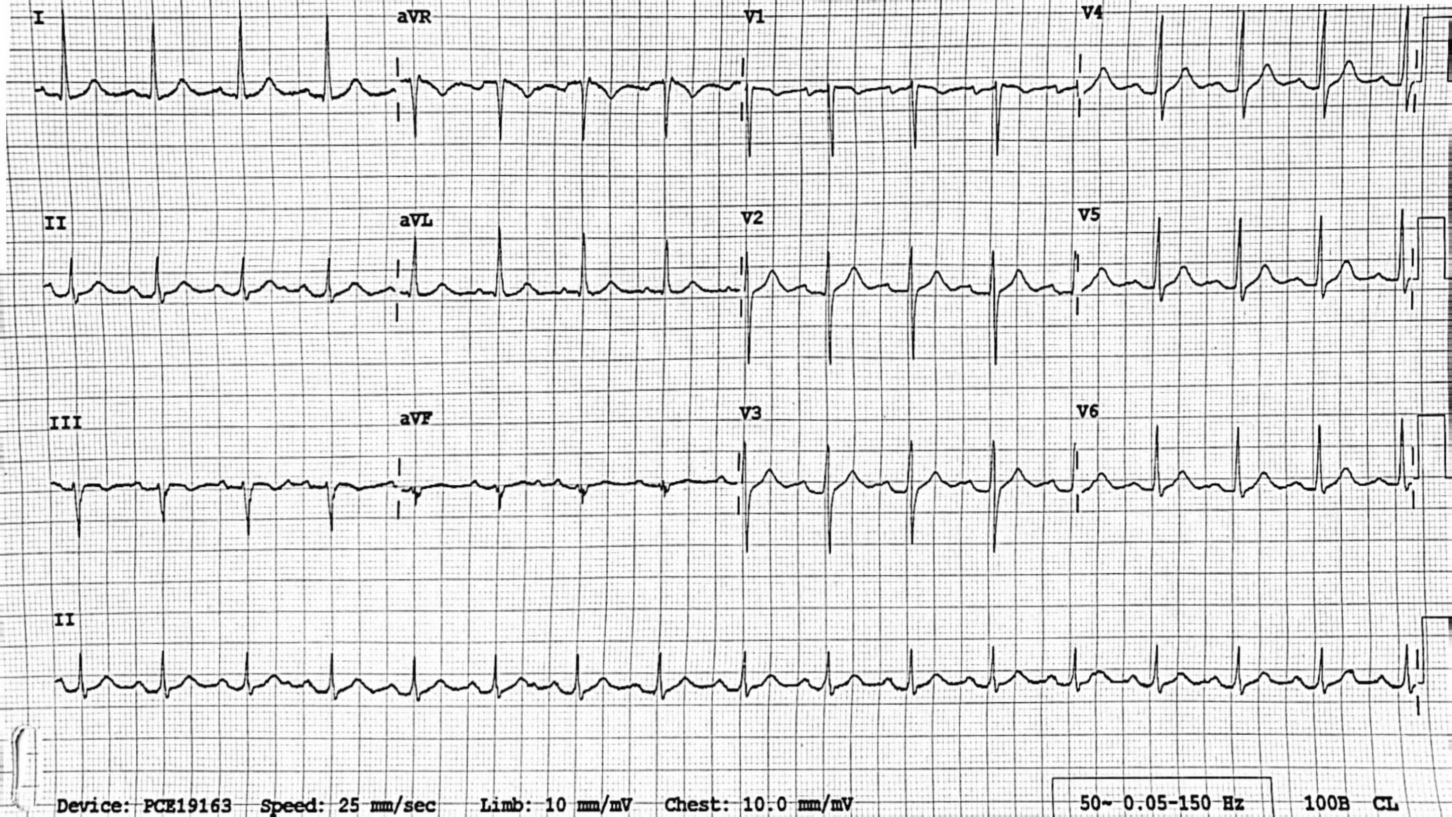
ECG during current hospital stay. ECG: electrocardiography.

On current admission, chest examination revealed crepitations at the right base (Figure 1). The rest of the systemic examinations were unremarkable. Elevated inflammatory markers and creatine kinase (CK) levels were noted, but ABG was normal. A chest X-ray indicated opacity in the right lower zone. His electrocardiogram (ECG) and bedside echocardiography revealed no evidence of cardiomyopathy (Figure 2). He was treated with intravenous (IV) co-amoxiclav 1.2 gm three times a day (TDS) and oral clarithromycin 500 mg BD, along with IV 0.9% sodium chloride (NaCl). His statin was discontinued due to rhabdomyolysis and due to the concurrent use of clarithromycin.

Upon admission, the patient had a blood glucose level of 27.8 mmol/L and was not eating or drinking adequately. Variable-rate intravenous insulin infusion (VRIII) was initiated to target capillary blood glucose (CBG) levels between 6 and 10 mmol/L. Blood and sputum cultures, as well as urinary antigens for Legionella and Pneumococcus, returned negative. Despite initial hydration with IV 0.9% NaCl, his CK levels did not decrease. Following a consultation with the metabolic unit (Charles Dent Metabolic Unit, University College London), IV hydration with 10% dextrose followed by 0.9% NaCl was initiated, along with strict monitoring of fluid balance, CBG, CK levels, and electrolytes. The patient's CBG levels remained stable throughout his hospital stay (Table 1).

**Table 1:** Blood investigations during current hospital stay.

Day	Creatinine kinase (CK) (40-320) U/L	C-reactive protein (CRP) (0-5) mg/L	White cell count (WCC) (4.3-11.2) 10^9^/L	Estimated glomerular filtration rate (eGFR) (90-200) mL/min	Bilirubin (0-21) umol/L	Alanine aminotransferase (ALT) (0-41) U/L	Alkaline phosphatase (ALP) (30-130) U/L	Gamma-glutamyl transferase (GGT) U/L
On admission	1628	100	19.4	44	23	35	81	-
1	39340	216	19	56	17	464	95	48
2	38977	180	-	57	-	-	116	-
3	15916	94	13.9	54	20	559	104	43
4	5030	55	11.8	51	20	472	83	46
5	2215	46	11.4	46	18	395	80	57
6	1215	35	11.6	49	17	314	80	64
7	626	20	10.7	54	14	247	78	62
8	388	13	10.1	51	14	202	77	70
9 days post-discharge	94	-	-	-	18	38	69	-

**Figure 3:**
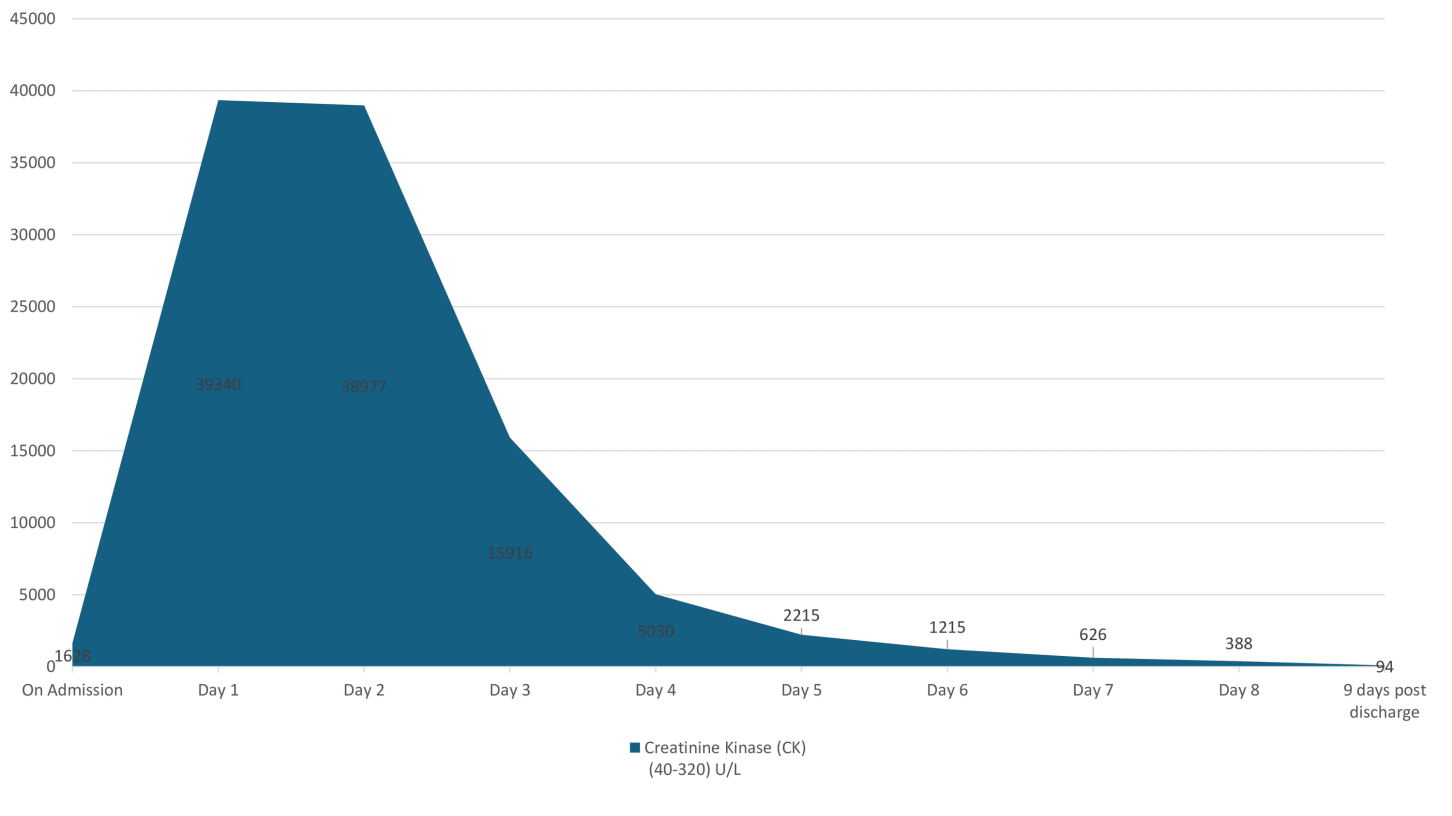
Progression of creatinine kinase (CK) levels during current illness.

At the time of discharge, nine days after hospitalization, his CK levels continued to improve. Two weeks after discharge, follow-up blood tests conducted by his general practitioner revealed normal results, including a CK level of 94 U/L (Figure 3).

## Discussion

CPT II deficiency is a hereditary metabolic disorder recognized as one of the most common causes of rhabdomyolysis and myoglobinuria [1]. It is also the most prevalent long-chain fatty acid oxidation defect [3]. The clinical presentations and severity depend on the level of residual enzyme activity [3]. There are three forms of CPT II deficiency: neonatal (6%), infantile (8%), and myopathic (86%) forms, all inherited in an autosomal recessive manner [4] caused by mutations in the CPT2 gene [5].

**Figure 4:**
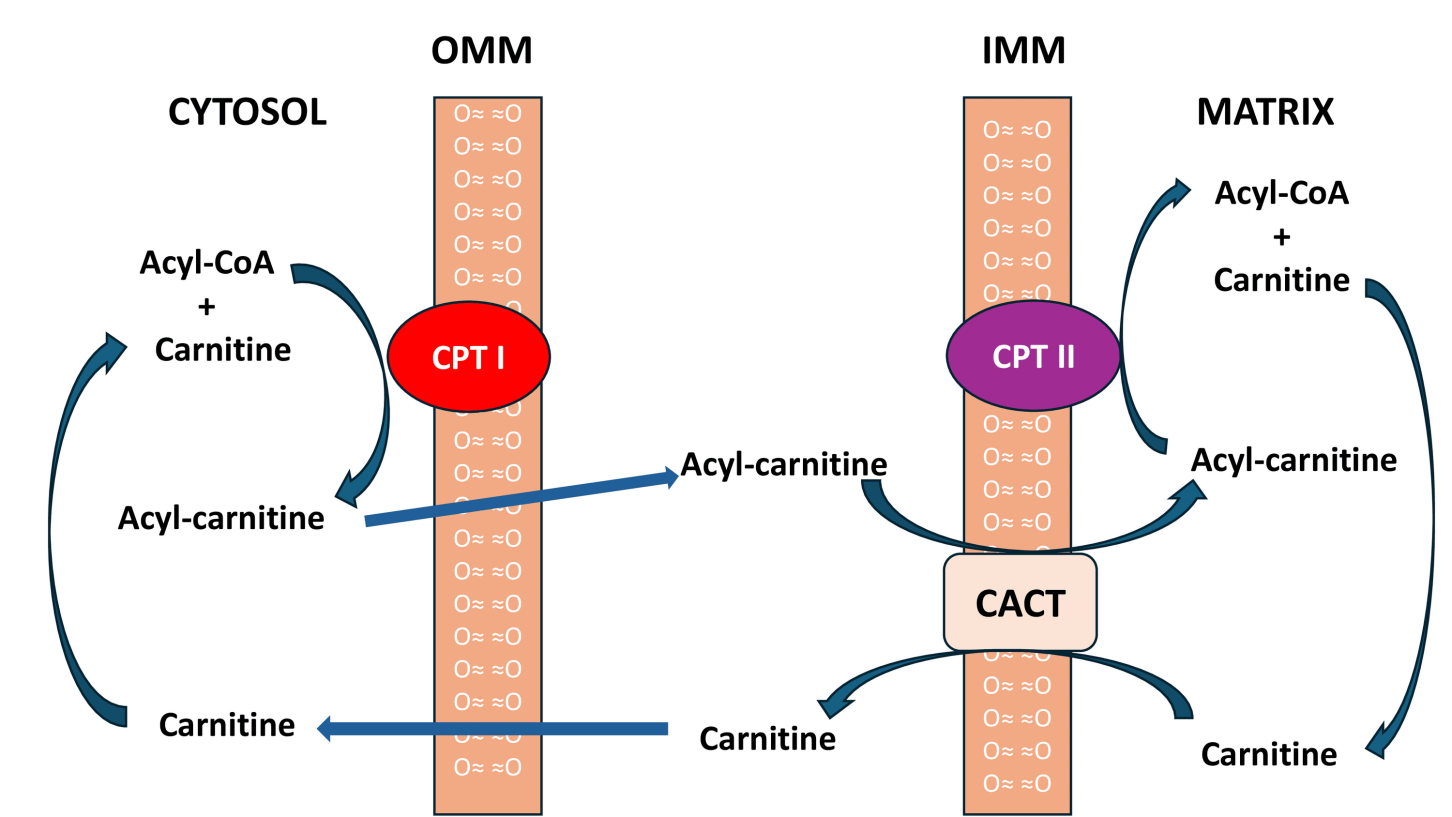
Transport pathway for esterification of fatty acids through mitochondrial membrane. OMM: outer mitochondrial membrane; IMM: inner mitochondrial membrane; CPT I: carnitine palmitoyltransferase 1; CPT II: carnitine palmitoyltransferase II; CACT: carnitine-acylcarnitine translocase. Note: This image is the author's own creation.

CPT II is in the inner mitochondrial membrane, whereas carnitine palmitoyltransferase 1 (CPT I) is in the outer mitochondrial membrane (Figure 4). CPT II functions with CPT I to transport the long-chain fatty acids from the cytosol into the mitochondria. CPT I initiates the process by converting long-chain acyl-CoA and carnitine into long-chain acylcarnitine and coenzyme A. This acylcarnitine is then transported across the intermembrane space by carnitine-acylcarnitine translocase (CACT) to the inner mitochondrial membrane, where CPT II splits it back into acyl-CoA and carnitine [6].

In myopathic CPT II deficiency, elevated calcium levels in the cytoplasm and mitochondria occur due to adenosine triphosphate (ATP) depletion and/or damage to the plasma membrane, leading to cytotoxicity [7]. Symptoms usually begin in childhood or early adulthood and include severe muscle pain and myoglobinuria triggered by extended physical exertion, viral illness, extremes of temperature, or fasting [7, 8]. Affected muscles will be tender and weak during attacks. Rhabdomyolysis can lead to complications such as renal failure and severe electrolyte imbalances, sometimes necessitating renal replacement therapy (RRT) [9]. Between attacks, there are typically no signs of myopathy, such as muscle weakness, pain, or elevated serum CK levels [9].

Myopathic CPT II deficiency shows a male predominance, with more than 75% of reported cases being male. The reason for this male predominance is uncertain, but may be related to differences in exercise patterns, an X-linked modifier gene, or hormonal factors like estrogen that could influence CPT regulation [7]. The diagnosis of CPT II deficiency is made by measurement of plasma acylcarnitine level followed by genetic studies for CPT2 gene mutation followed by measurement of CPT II enzymatic activity [5].

The treatment strategy for acute rhabdomyolysis in CPT II deficiency involves three main components: administering IV glucose, managing rhabdomyolysis, and discontinuing any medications that may exacerbate the condition [9]. Initial acute treatment focuses on providing adequate glucose to prevent lipolysis and to prevent the hypoglycemia that is typically associated with acute episodes. IV glucose should not be delayed even if blood glucose levels appear normal. It is suggested to initiate intravenous 10% dextrose at a rate of 2 ml/kg/hr (e.g., 140 ml/hr for a 70 kg person). Always aim to achieve CBG between 6 and 10 mmol/L. In the event of CBG >10 mmol/L, consider starting VRIII rather than reducing the 10% dextrose infusion rate. Hypoglycemia should be treated with 50 ml of 50% dextrose over 30 minutes. Additionally, dehydration should be corrected with 0.9% NaCl and serum potassium levels should be monitored and corrected appropriately [10].

Managing rhabdomyolysis involves administering intravenous crystalloid fluids, typically 0.9% NaCl with or without Ringer's lactate, to achieve a urine output of 200 to 300 mL per hour. If systemic acidosis is present, sodium bicarbonate should be given to raise urine pH above 6.5 to reduce myoglobin-induced kidney damage. However, evidence supporting the benefit of urinary alkalization over hydration alone in preventing AKI is limited [11]. Dialysis should be considered in the event of significant renal impairment [10].

A thorough medication review should be performed, including all prescribed medications, over-the-counter drugs, and herbal remedies. It is essential to avoid medications that could trigger or worsen muscle injury, both in acute and non-acute settings. Medications to avoid include statins (which increase the risk of rhabdomyolysis), ibuprofen, and valproic acid [9]. Additionally, the underlying cause of the rhabdomyolysis, such as an infection or other clinical issue, should be managed appropriately.

In this case report, we describe a patient with CPT II deficiency who presented with a chest infection that precipitated rhabdomyolysis. The patient’s medical history includes a significant prior ITU admission for severe sepsis from bilateral pneumonia complicated by rhabdomyolysis and acute kidney injury. Elevated inflammatory markers and CK levels were noted. After administering targeted IV fluids and antibiotics, his CK levels normalized, and he was discharged in a stable condition.

This case highlights several important learning points in the management of CPT II deficiency complicated by rhabdomyolysis. Early recognition of infections as potential triggers for rhabdomyolysis is crucial in preventing severe complications. Targeted IV fluid therapy (10% dextrose to avoid catabolism and 0.9% NaCl for rehydration) plays a vital role in managing rhabdomyolysis and preventing AKI. Regular medication reviews are necessary to avoid drugs that could trigger or worsen rhabdomyolysis in patients with metabolic myopathies. Additionally, prompt identification and treatment of infections are essential to minimize muscle breakdown. Finally, vigilant monitoring and proactive care are critical for patients with a history of severe complications, such as previous ITU admissions, to prevent recurrence and ensure better outcomes.

## Conclusions

CPT II deficiency, a genetic metabolic disorder affecting long-chain fatty acid metabolism, often presents with intense muscle pain, muscle weakness, myoglobinuria and rhabdomyolysis, worsened by physical exertion, infection, fasting, or extremes of temperature. Elevated calcium levels resulting from ATP depletion and membrane damage can lead to severe complications, such as renal failure and electrolyte disturbances. Effective management includes prompt IV glucose and fluid therapy, rigorous medication review, and addressing underlying triggers. This case highlights the importance of proactive monitoring and treatment to avoid severe complications and ensure patient well-being.
